# A combined activity of peripheral nociceptive and nonnociceptive neurons is necessary to trigger spinal microglial reactivity and sustained pain

**DOI:** 10.1097/j.pain.0000000000003725

**Published:** 2025-07-23

**Authors:** Manon Isler, Paul Chu Sin Chung, Alexandru-Florian Deftu, Guylène Kirschmann, Isabelle Decosterd, Marc Rene Suter

**Affiliations:** a Pain Center, Department of Anesthesiology, Lausanne University Hospital (CHUV), Lausanne, Switzerland; b Department of Fundamental Neurosciences, Faculty of Biology and Medicine, University of Lausanne, Lausanne, Switzerland

**Keywords:** Pain, Electrical stimulation, Optogenetic, DRG neurons, Spinal microglia

## Abstract

Abnormal activity in injured nerve fibers triggers spinal microglial reactivity, a critical step in the development of chronic postoperative pain. It remains unclear whether spinal microglia respond to inputs from nociceptive fibers alone or require combined activity from nociceptors and nonnociceptors. Using electrical and optogenetic approaches, we selectively activated different fiber subtypes without causing neuronal injury. Our findings demonstrate that only simultaneous activation of both nociceptive and nonnociceptive fibers induces sustained hypersensitivity and a spinal microglia response. This response is characterized by increased cell proliferation, altered electrophysiological profile with hyperpolarized membrane potential, increased potassium currents, and a less ramified morphology. In contrast, activation of nociceptive or nonnociceptive fibers alone does not induce these changes. In addition, inhibiting spinal microglia with minocycline prevents those changes. Overall, our study suggests that both types of peripheral sensory input are necessary to elicit microglial reactivity in the spinal cord and the subsequent development of sustained pain-related behavior.

## Abstract 2

Supplemental Digital Content is Available in the Text.

Spinal microglia become reactive and trigger hypersensitivity in response to combined optogenetic activation of nociceptive and nonnociceptive fibers.

## 1. Introduction

Chronic postoperative pain, often of neuropathic origin, is a debilitating condition affecting 5% to over 85% of patients, depending on the surgery type.^32,45,57^ Current treatments are frequently ineffective and carry adverse side effects.^47,80^ Therefore, there is an urgent need for a better understanding of the underlying mechanisms to develop preventive interventions.

Altered excitability in primary sensory neurons (PSN) after nerve injury^2,19,31,33,44,68^ has been shown to be a critical factor in chronic pain onset.^14,25,43,56,61,70,76^ It involves increased spiking, both evoked and spontaneous. In addition, microglia, immune cells of the central nervous system, play a crucial role in chronic pain initiation.^5,9,10,16,27,52,75^ Blocking microglial activity or depleting microglia early after peripheral nerve injury (PNI) can prevent chronic pain development.^26,28,40,41,46,48,53,54,74^ In the dorsal horn of the spinal cord, microglia react to inputs from injured neurons by proliferating, changing morphology, initiating inflammatory responses, and altering their electrophysiological properties.^21–23,34,39,73^

Both hyperexcitability in peripheral afferents and spinal microglia reactivity are necessary for chronic neuropathic pain onset and seem to be interconnected. In neuropathic pain models, blocking all PSN types, ie, nociceptive and nonnociceptive fibers and motor neurons from the sciatic nerve, before injury prevents spinal microglial reactivity. However, selective blockade does not yield the same results.^64,71^ This suggests that microglial reactivity is directly linked to PSN activity and depends on the subtype of sensory fibers.

Few studies have shown that neuronal activity from different PSN subpopulations is sufficient for spinal microglial reactivity and chronic pain. One study^30^ demonstrated that electrical stimulation of the sciatic nerve at an intensity recruiting both unmyelinated C fibers and myelinated Aβ/Aδ fibers triggers microglial proliferation, inflammatory responses, and hypersensitivities in male rats. C fibers and Aδ fibers are classically considered nociceptive, whereas Aβ fibers are traditionally viewed as nonnociceptive. Lower intensity stimulation, recruiting only Aβ and Aδ fibers, failed to induce similar reactivity. Importantly, no neuronal injury was induced, indicating that neuronal activity is sufficient to drive microglial responses. However, recent classification based on single-cell RNA-sequencing identified 11 subtypes of PSN neurons^66^ and demonstrated that classifying nociceptors vs nonnociceptors solely based on fiber type is oversimplistic, as C and Aδ fibers are not exclusively nociceptive. Thus, it remains unclear if nociceptive inputs alone are sufficient to produce spinal microglial reactivity and persistent pain or if a combined nociceptive and nonnociceptive activity is necessary.

Therefore, our goal was to investigate which neuronal peripheral inputs are responsible for spinal microglial reactivity and for persistent pain.

We first confirmed that peripheral electrical stimulation elicited spinal microglial reactivity and pain in mice, as previously observed in rats. Then, using an optogenetic approach, we activated different subtypes of primary sensory neurons in the sciatic nerve (all PSN subtypes, nociceptive subtypes or nonnociceptive neurons alone). We evaluated spinal microglial responses and pain-related behaviors after these activations. Finally, we investigated the role of spinal microglia in the development of pain hypersensitivity after electrical stimulation by using minocycline, an antibiotic known to inhibit microglia.

## 2. Material and methods

All detailed references for reagents and materials can be found in the reagents table.

### 2.1. Study approval

All experiments were done in accordance with the Swiss Federal Animal Protection Law and the guidelines provided by US National Institutes of Health guidelines^29^ and the International Association for the Study of Pain (IASP).^81^ The project was approved by the Committee on Animal Experimentation for the canton of Vaud, Switzerland (license VD3068.2b).

### 2.2. Animals

All animals were grouped housed and kept at a constant temperature with a 12 hours/12 hours dark/light cycle and ad libitum access to food and water. Adult mice from 8 to 12 weeks were used for all experiments. Heterozygote B6.129P-Cx3cr1tm1Litt/J, referred to as CX3CR1-GFP, and C57BL/6J (Charles-Rivers Laboratories, Wilmington, MA) were used for electrical stimulation. Optogenetic stimulations were done on heterozygous Scn10a (SNS)-Cre/ChR2-TdTomato, Advillin-Cre/ChR2-TdTomato, Ntng1-Cre/ChR2-TdTomato, referred to as SNS-ChR2, Advillin-ChR2, Ntng1-ChR2, and control littermates. These lines were obtained by crossing B6.Cg-Gt (ROSA) 26Sortm27.1 (CAG-COP4*H134R/tdTomato) Hze/J from Jackson Laboratory and C57BL/6-Tg (Scn10a::Cre)1Rkun line,^1^ B6; D2-Tg (Avil-cre) 1Phep/Cnrm,^82^ and Ntng1-Cre.^6^ SNS-ChR2 targets primarily nociceptive neurons, Advillin-ChR2 broadly targets primary sensory neurons (both nociceptive and nonnociceptive), and Ntng1-ChR2 predominantly targets nonnociceptive neurons. The selection of these promoters was guided by Table 1 of Santana-Varela et al.,^58^ which summarizes single-cell RNA sequencing data from earlier studies to characterize dorsal root ganglia (DRG) neuronal subtypes.

### 2.3. Electrical stimulation

Electrical stimulation of the sciatic nerve was done on CX3CR1-GFP and C57BL/6J mice under general anesthesia (2% isoflurane). The sciatic nerve was exposed by opening the fascial plane between the gluteus maximum and the anterior head of the biceps femoris to avoid incision through the muscles. The sciatic nerve was dissected free of perineural membranes. A small piece of nitrile glove was placed under the sciatic nerve to electrically isolate it from the surrounding muscle. Great care was taken to avoid stretching the nerve. A home-made bipolar electrode, consisting of 2 silver wires placed approximately 1 to 2 mm apart, was gently placed on the exposed sciatic nerve and connected to a current stimulator (DS3 Isolated Current Stimulator). As a bipolar configuration provides both stimulation poles, no additional ground electrode was required. The current stimulator was driven by an electronic stimulator (Grass SD9 B Square Pulse Stimulator), delivering square monophasic pulses at 3 mA, 500 μs pulse duration, 10 Hz, for 5 minutes (adapted from Hathway et al.^30^).

### 2.4. Optogenetic stimulation

Optogenetic activation of the sciatic nerve was performed on SNS-Cre/ChR2-TdTomato, Avil-Cre/ChR2-TdTomato, Ntng1-Cre/ChR2-TdTomato, and their control littermates. The surgical approach was similar to the electrical stimulation protocol. Blue light (470 nm) was then applied above the sciatic nerve using a pulsing laser system (LRD-0470 Collimated Diode Laser System) and a split optic fiber (2 m long, optical Fiber Core Diameter = 200 μm, NA = 0.37) to stimulate 2 mice at the same time. To ensure that the laser was at the same distance (approximately 12 mm) from the sciatic nerve between each animal, the laser was kept in place in a small 3D-printed plastic funnel that was placed above the hindlimb of the stimulated mice. The stimulation was done for 30 minutes with the following parameters 2 Hz, 50 milliseconds pulse duration, 5 mW/mm^2^. An Arduino board was used to drive the whole optogenetic system with the chosen stimulation parameters.

### 2.5. Behavior

Mechanical and thermal sensitivities were assessed with Von Frey and Hargreaves test, respectively. The experimenter was blinded to genotypes of the animals. Mice were habituated to the experimental setups and to the experimenter handling for 1 week before the experimental procedures. On each testing day, they were allowed 30 additional minutes of habituation to the room and setups.

#### 2.5.1. Von Frey

Mice were confined in polymethyl methacrylate boxes (7 cm × 6.5 cm × 5 cm) on an elevated mesh grid platform. Von Frey filaments ranging from 0.02 to 2.0 g were applied using the up-and-down method,^7,13^ starting with 0.16-g filament. Filaments were applied on the lateral part of the plantar hind paws. If the filament elicited a withdrawal response, the lighter filament was applied. If no response was elicited, the following heavier filament was applied. Maximum 6 stimulations were applied on each paw, and the withdrawal threshold (in grams) was calculated based on the response sequence.

#### 2.5.2. Hargreaves

Mice were tested in polymethyl methacrylate boxes placed on a transparent glass floor set at 30°C. The lateral surface of the plantar hind paws was exposed to a beam of radiant heat, with a cutoff set at 20 seconds (IITC Life Science Inc, Woodland Hills, CA). The latency to paw withdrawal was measured 3 times on each paw, with at least 10 minutes between each stimulation.

### 2.6. Minocycline

For the microglial inhibition experiment, heterozygous CX3CR1-eGFP mice were randomly attributed to vehicle or minocycline groups. Minocycline was diluted in 500-μL saline solution. Vehicle or minocycline (30 mg/kg) were intraperitoneally injected in heterozygous CX3CR1-eGFP mice (n = 10 per group) 1 hour before electrical stimulation, and then every 24 hours until 2 days after the stimulation.

### 2.7. Immunofluorescence

Animals were terminally anesthetized with pentobarbital (50 mg/kg) and transcardially perfused with saline followed by ice-cold paraformaldehyde (PFA) (4% wt/vol in PB 0.1M). Ipsilateral DRGs (L3, L4, and L5) and lumbar spinal cord were collected, postfixed in PFA 4%, and cryoprotected in 20% sucrose overnight. Tissues were frozen in optimal cutting temperature medium and cut in series using a cryostat. For immunofluorescence quantification, spinal cord tissue was cut into 20-μm thick transversal sections. DRGs were cut into 12-μm thick sections. For immunofluorescence staining, slides were washed in phosphate buffered saline (PBS) 1X, then blocked with 10% serum (normal horse or normal goat serum) in PBS 1X + 0.3% Triton for 30 minutes at room temperature. They were then incubated overnight at 4°C with the following primary antibodies diluted in 5% serum in PBS 1X + 0.1% Triton: goat anti-Iba1 (1:500, Abcam, Waltham, MA), rabbit anti-Ki67 (1:500, Abcam), rabbit anti-ATF3 (1:500, Abcam), mouse anti-NF200 (1:200, Sigma-Aldrich Inc., Saint-Louis, MO) or rabbit anti-NF200 (1:100, Sigma-Aldrich Inc), rabbit anti-peripherin (1:2000, Sigma-Aldrich Inc), rabbit anti-CGRP (1:5000, Peninsula Laboratories International Inc., San Carlos, CA), and biotinylated anti-IB4 (1:50, Vector Laboratories, Newark, CA). After washing 3 times for 5 minutes with PBS 1X, slides were incubated with the following secondary antibodies diluted in 1% serum in PBS 1X + 0.1% Triton: Alexa Fluor 488–conjugated donkey antigoat (1:500, Molecular Probes, Eugene, OR), Cy3-conjugated donkey antigoat (1:300, Jackson ImmunoResearch, Ely, United Kingdom), Cy5-conjugated goat antirabbit (1:500, Invitrogen, Waltham, MA), Alexa Fluor 647 conjugated donkey antirabbit (1:500, Invitrogen), Alexa Fluor 488–conjugated donkey antirabbit (1:500, Molecular Probes), Alexa Fluor 488–conjugated goat antimouse (1:200, Molecular Probes), Alexa Fluor 350–conjugated goat antirabbit (1:2000, Molecular Probes), and AMCA-conjugated Streptavidin (1:200, Jackson ImmunoResearch). The slides were again washed 3 times in PBS. For 4',6-diamidino-2-phenylindole (DAPI) staining, DAPI was diluted to 300 nM in 1% serum in PBS 1X + 0.1% Triton and applied for 5 minutes. The slides were washed 3 additional times in PBS and then mounted using Mowiol mounting medium.

For microglial morphological analysis, spinal cords were cut in 40-μm thick transversal sections in free-floating method and were kept in a cryoprotectant buffer (25% Glycerol, 30% Ethylene Glycol, 45% PBS 1X). The sections were then stained using the protocol described above while agitated and then mounted on slides.

### 2.8. Image acquisition and analysis

For immunostaining quantification, images were acquired with a 20X objective on a Zeiss Axioscan Z.1. Quantification was done using Zeiss Zen3.3 (blue edition) and Fiji Software. The quantification was done on several sections per mouse (spinal cord, 10-15 sections; DRGs, 4-6 sections; from 6 to 7 mice in each group) and averaged. For the spinal cord, the region of interest (ROI) was defined by a rectangle drawn to cover the dorsal horn. For the DRG, the region of interest was defined as the region comprised of neuronal cell bodies. The number of counted cells was normalized to the area of ROI and expressed as the number of cells per mm^2^. The experimenter was blinded to the genotype and stimulation protocol.

For morphological analysis of microglial cells after stimulation, images were acquired on the ipsilateral dorsal horns of 40-μm thick slices using a Leica Stellaris 8 confocal microscope (Leica, Wetzlar, Germany). DAPI, Iba1, and Ki67 immunofluorescence were acquired. The microscope was set to line sequence, with a 2048 × 2048 resolution, a laser speed set to 400, and a line average set to 4. In z axis, images were acquired for approximately 30 μm. The Z-step size was 0.30 μm, and optical section was 0.896 μm. Images were then extracted as multipage Tagged Imaged File Format(TIFF) files and preprocessed using ImageJ adjust threshold command. For each file, the threshold was set between 20 and 30 depending on the quality of the staining. As Iba1 expression is mainly cytoplasmic, the threshold was set to ensure the soma of microglial cells stained against the Iba1 antibody was visible for further morphological analysis. Noise was reduced by removing outliers (2.0 pixel radius, threshold 50). The resulting tiff files were analyzed using published MATLAB script from Brian A. MacVicar's lab.^77^ Briefly, a threshold at 0.3 and a noise filtering between 20′000 and 30′000 were applied to every image. Then, original cells, skeleton, branchpoint, and endpoints were calculated using 1 of the 2 available methods (“Keep small processes”). A table was generated at the end of the analysis and contained the following values for each individual cell: cell territory volume, cell volume, and Ramification Index (cell territory volume divided by cell volume). The cell territory volume corresponds to the volume surveilled by the cell and is computed based on a polygon drawn around the endpoints of the cell. Results were then manually checked to ensure that the script properly recognized the soma of the analyzed cells. These values were then compared between groups. The experimenter was blind to genotypes for the whole procedure.

### 2.9. Microglia primary culture

CX3CR1-eGFP mice were terminally anesthetized by intraperitoneal injection of pentobarbital and were then decapitated. The spinal column was removed, and the lumbar spinal cord was flushed out from the sacral part using PBS. The tissue was transferred to a petri dish containing PBS. Under a stereomicroscope (Leica MZ6), the ipsilateral spinal cord dorsal horn (SCDH) was isolated, transferred into a petri dish containing Dulbecco's Modified Eagle Medium (DMEM) with papain (2 mg/mL). Using a scalpel, the tissue was chopped to pieces and then incubated on a shaker for 30 minutes at 30°C. Using a micropipette, the medium with papain was discarded and replaced with 1 mL of DMEM supplemented with 10% heat inactivated fetal bovine serum (FBS) and 1% penicillin–streptomycin (P/S) and allowed to settle for 2 minutes. The medium containing the cells was transferred to a 15-mL falcon tube. Mechanical trituration using a micropipette was done. The procedure was repeated twice. The resulting 3 mL of cell suspension was then centrifuged at 1500 rpm for 5 minutes at room temperature. The supernatant was discarded. The cells were resuspended in DMEM + 10% FBS + 1% P/S (30 μL/coverslips) and then plated on coverslips in 24 well-plates. After 1 hour of incubation at 37°C and 5% CO2, 500 μL of medium (DMEM + 10% FBS + 1% P/S) was added per well. After another 1 hour of incubation, the medium was replaced with fresh 1. Patch-clamp recordings were done on the following day.

### 2.10. Whole-cell patch-clamp recordings

CX3CR1-eGFP^+^ microglia from dissociated L3-L5 lumbar spinal cord were patched in whole-cell configuration. We analyzed data from microglia that had an access resistance < 20 MΩ and a hold > −15 pA when clamped at −60 mV in voltage-clamp mode. Resting membrane potential was measured in 1-minute-long current-clamp recording in extracellular solution. The voltage-clamp protocol consisted of 500-millisecond-long steps from −160 to +40 mV, increasing in 10-mV increments. Recorded cells originated as follows: for resting membrane potential in current-clamp mode, 37 cells from 4 sham mice and 37 cells from 3 stimulated mice were recorded. From these cells, 33 cells from sham and 29 cells from stimulated group went through the entire voltage steps protocol, the remaining cells were lost during the acquisition.

#### 2.10.1. Solutions

Patch-clamp recordings were made in extracellular solution containing (in mM): 120 NaCl, 20 KCl, 2 CaCl_2_ × 2H_2_O, 1 MgCl_2_ × 6H_2_O, 10 HEPES, 10 D-glucose. pH was adjusted to 7.4 with NaOH at room temperature, and osmolarity was adjusted to 305 to 310 mOsm/kg. Intracellular solution contained (in mM): 5 NaCl, 130 KCl, 1 CaCl_2_ × 2H_2_O, 2 MgCl_2_ × 6H_2_O, 10 4-(2-hydroxyethyl)-1-piperazineethanesulfonic acid (HEPES), and 11 ethylene glycol-bis(β-aminoethyl ether)-N,N,N',N'-tetraacetic acid. pH was adjusted to 7.35 with potassium hydroxide, and osmolarity was adjusted to 230 to 300 mOsm/kg.

#### 2.10.2. Data acquisition

Fire-polished borosilicate glass pipettes (BF150-86-7.5HP, Sutter Instruments Co, Novato, CA) were pulled using a micropipette puller (model P-97, Sutter Instruments Co) and fire-polished using a MF200-2 microforge, H4 platinum/iridium wire (World Precision Instruments, Sarasota, FL), and W30S-LED Revelation III to a resistance near 5 MΩ. Data were acquired using a MultiClamp 700B amplifier and a Digidata 1440A driven by pClamp 10.3 software (Molecular Devices LLC, San Jose, CA). The whole-cell recordings were made suing a BX51W1 microscope (Olympus Corporation, Tokyo, Japan) equipped with a ORCAFlash 2.8 digital camera (Hamamatsu Photonics, Hamamatsu City, Japan) driven by CellSens v3.2 acquisition software. Microglia were selected based on their eGFP expression using a CoolLED pE-340fura and a LED eGFP pE-300 filter set. The fast, slow, and whole-cell compensations were made with Multiclamp 700B at a bandwidth of 5 kHz and a low-pass filter of 10 kHz.

### 2.11. Statistical analysis

The full statistical analyses performed are presented in the DATA table and were performed using GraphPad Prism 10 software. In the box plots, the center lines indicate the median, the plus signs indicate the mean, and the box limits indicate the upper and lower percentiles (5%-95%). Line graphs are represented as mean ± SD. Two-way ANOVA were used for patch-clamp I–V curve analysis with Voltage Steps/Group/Interaction factors and for histological analysis with Dorsal horn side/Group/Interaction factors. For behavioral analysis, 2-way ANOVA with repeated measures were used with Group/Time/Genotype/Interaction factors. In case of a significant statistical effect, a post hoc multicomparison analysis using either Tukey or Sidak correction was applied. For 2 groups comparisons, normality was assessed by D'Agostino & Pearson test, and an unpaired 2-tailed *t* test or Mann–Whitney test was then applied accordingly. The threshold for statistical significance was set at *P*-value <0.05. The thresholds were represented as follows: *P ≤ 0.05, **P ≤ 0.01, ***P ≤ 0.001, and ****P ≤ 0.0001.

## 3. Results

### 3.1. Dorsal horn spinal microglia react to electrical stimulation of sciatic nerve without nerve injury

**Figure 1.**
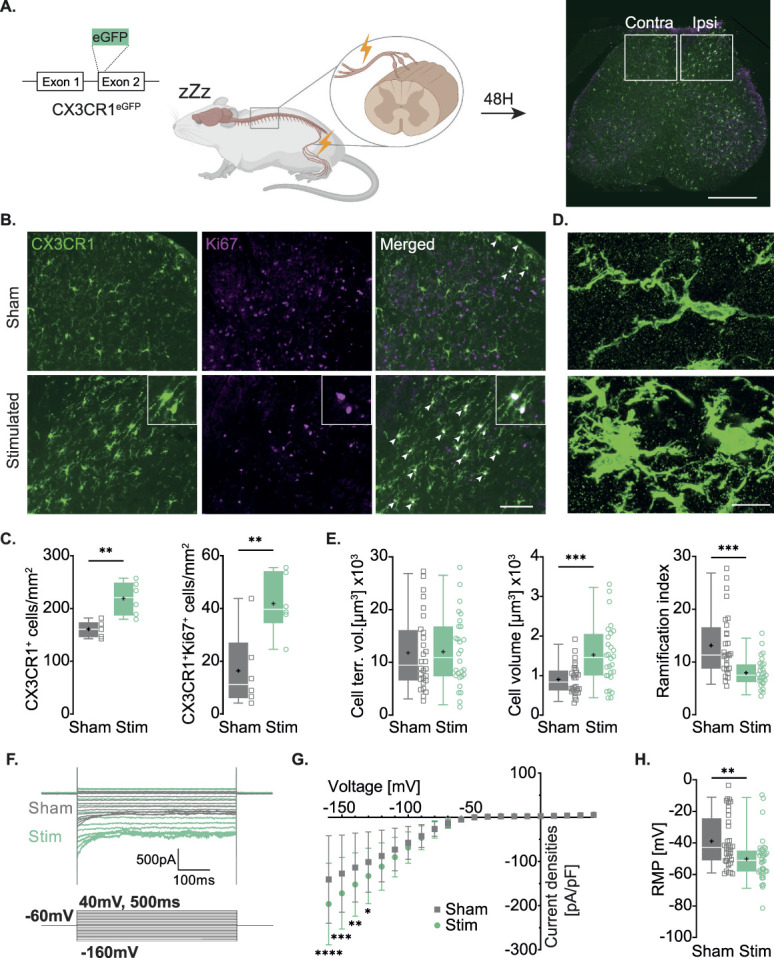
Electrical stimulation of the sciatic nerve induces spinal microglial reactivity. (A) Experimental design and representative spinal cord 2 days after electrical stimulation. Mice received either sham (n = 6) or electrical stimulation (n = 6). Analysis was done in contralateral and ipsilateral SCDH (white rectangles). Scale bar 500 μm. (B) Representative images of ipsilateral SCDH with CX3CR1^+^ cells (microglia, in green), Ki67^+^ (proliferating cells, in purple), and merged (proliferating microglia, in white). Arrowheads point to colocalization examples. Scale bar 100 μm. (C) Left. Number of CX3CR1-eGFP^+^ cells per mm^2^. Mean ± SD: Sham = 160.8 ± 14.7, Stim = 219.2 ± 31.6. 2W ANOVA (Dorsal horn side *P* = 0.0007, Group *P* = 0.0028, Interaction *P* = 0.0228) with Tukey multiple comparisons post hoc (*P* = 0.0025). Right. Number of CX3CR1-eGFP^+^ Ki67^+^ cells per mm^2^. Mean ± SD: Sham = 16.4 ± 14.7, Stim = 41.9 ± 11.5. 2W ANOVA (Dorsal horn side *P* < 0.0001, Group *P* = 0.0033, Interaction *P* = 0.0037) with Tukey multiple comparisons post hoc (*P* = 0.0008). (D) Representative images of ipsilateral SCDH microglial morphology of sham (top) and stimulated (bottom) mice. Scale bar 10 μm. (E) Quantification of SCDH microglia morphology from sham (n = 28 cells from 3 mice) and Stim (n = 28 cells from 3 mice) group. Left. Cell territorial volume. Mean ± SD: Sham = 11,811 ± 7079, Stim = 12,027 ± 6744. Unpaired t test, 2 tailed, *P* = 0.9075. Middle. Cell volume. Mean ± SD: Sham = 901.4 ± 385.1, Stim = 1521.7 ± 770.2. Unpaired t test, 2 tailed, *P* = 0.0004. Right. Ramification index. Mean ± SD: Sham = 13.1 ± 6.0, Stim = 7.9 ± 2.8. Unpaired t test, 2 tailed, *P* = 0.0001. (F) Representative traces of currents of dissociated SCDH microglia from sham (grey) and stimulated mice (green) in response to voltage steps from −160 mV to 40 mV with 10 mV increments for 500 ms. Voltage steps protocol on bottom. (G) I–V curve showing current densities [pA/pF] of dissociated SCDH microglia from sham (n = 33) and stimulated mice (n = 29). Mean ± SD (at −160 mV): Sham = −140.7 ± 99.1, Stim = −196.3 ± 92.5. 2W RM ANOVA (Voltage steps *P* < 0.0001, Groups *P* < 0.0001, Interaction *P* < 0.0001) with Sidak multiple comparisons post hoc. (H) Resting membrane potential (in mV) of SCDH microglia from sham (n = 37) and stimulated mice (n = 37). Mean ± SD: Sham = −38.9 ± 16.3, Stim = −50.1 ± 14.1. Mann-Whitney test, *P* = 0.0026. Contra, contralateral; Ipsi, ipsilateral; RMP, resting membrane potential; Stim, stimulated.

First, we asked whether electrical stimulation on the sciatic nerve recruiting both nociceptive and nonnociceptive neurons would induce SCDH microglial reactivity in mice, similar to what was previously shown in rats. Two days after sciatic nerve stimulation in the CX3CR1-eGFP mouse line (Fig. 1A), we observed an increased number of GFP^+^ cells in the ipsilateral SCDH of stimulated mice compared to sham mice (Fig. 1B, C left, Sham = 160.8 ± 14.7 cells/mm^2^, Stim = 219.2 ± 31.6 cells/mm^2^). In line with this result, we also observed an increased proliferation when quantifying Ki67^+^ cells in this population in ipsilateral SCDH of stimulated mice compared to sham mice (Fig. 1C right, Sham = 16.4 ± 14.7 cells/mm^2^, Stim = 41.9 ± 11.5 cells/mm^2^). No changes were observed between the groups in the contralateral SCDH (see statistical table, http://links.lww.com/PAIN/C328).

We found no difference in the number of ATF3^+^ neurons in the averaged L3, L4, and L5 ipsilateral DRGs compared to the sham group (Fig. S1 A, B, http://links.lww.com/PAIN/C330, Sham = 106.6 ± 59.7 cells/mm^2^, Stim = 185.0 ± 81.8 cells/mm^2^). Moreover, we found no change in the number (Fig. S2 B, http://links.lww.com/PAIN/C330 top, Sham = 357.7 ± 91.4 cells/mm^2^, Stim = 389.4 ± 87.6 cells/mm^2^) and proliferation (Fig. S2 B, http://links.lww.com/PAIN/C328 bottom, Sham = 2.7 ± 3.3 cells/mm^2^, Stim = 4.6 ± 3.7 cells/mm^2^) of CX3CR1-eGFP^+^ macrophages in the DRGs.

We then investigated the morphological changes of spinal microglia after stimulation (Figs. 1D and E) using a published MATLAB script.^77^ The cell territorial volume of ipsilateral SCDH microglia was unchanged (Fig. 1E left, 11,811 ± 7079 μm^3^, Stim = 12,027 ± 6744 μm^3^). However, we observed an increased cell volume (Fig. 1E middle, Sham = 901.4 ± 385.1 μm^3^, Stim = 1521.7 ± 770.2 μm^3^) and a decreased ramification index (Fig. 1E right, Sham = 13.1 ± 6.0, Stim = 7.9 ± 2.8) from stimulated group compared to sham.

Finally, we used the whole-cell patch-clamp technique to measure electrophysiological changes in dissociated spinal microglia 2 days after sham or electrical stimulation. When applying a voltage-steps protocol (Fig. 1F), our recordings reveal increased inward currents from −160 mv to −130 mV compared to controls (Fig. 1G, Sham at −160 mV = −140.7 ± 99.1 pA/pF, Stim at −160 mV = −196.3 ± 92.5 pA/pF). Cells were also hyperpolarized compared to controls (Fig. 1H, Sham = −38.9 ± 16.26 mV, Stim = −50.13 ± 14.1 mV).

### 3.2. Anatomical characterization of Advillin-, SNS-, and Ntng1-ChR2 mouse line

Once we confirmed that electrical stimulation of the sciatic nerve at an intensity recruiting both nociceptive and nonnociceptive fibers could induce spinal microglial reactivity, we wanted to gain specificity when activating the subpopulations of neurons. Thus, we crossed different Cre lines to a floxed ChR2-TdTomato line to be able to optogenetically activate the different populations of primary sensory neurons. We used the Advillin-ChR2 line to target both nociceptive and nonnociceptive neurons, the SNS-ChR2 line to target nociceptive neurons, and finally, the Ntng1-ChR2 line to target nonnociceptive neurons. We first characterized the expression of the channelrhodopsin (ChR2-TdTomato^+^ neurons) in the DRGs. To do so, we immunostained DRG sections against A-fiber (mostly nonnociceptive) neuronal marker Neurofilament Protein (NF200), C-fiber (mostly nociceptive) neuronal marker Peripherin, peptidergic nociceptive neuronal marker Calcitonin Gene–Related peptide (CGRP), and nonpeptidergic nociceptive neuronal marker Isolectin B4 (IB4).

Overall, we found that 447.6 ± 35.9 cells per mm^2^ were ChR2-TdTomato^+^ in the Advillin-ChR2 mice (Fig. S3 B, http://links.lww.com/PAIN/C330). Among those ChR2-TdTomato^+^ cells, we found that 41.5% were myelinated A fibers (NF200^+^, Fig. S3 C, D, http://links.lww.com/PAIN/C330) and 39.2% were unmyelinated C fibers (Peripherin^+^, Fig. S3 E, F, http://links.lww.com/PAIN/C330), with 16.6% being peptidergic (CGRP^+^, Fig. S3 G, H, http://links.lww.com/PAIN/C330) and 9.2% nonpeptidergic (IB4^+^, Fig. S3 I, J, http://links.lww.com/PAIN/C330).

In the SNS-ChR2 line, 612.2 ± 45.7 cells per mm^2^ were ChR2-TdTomato^+^ in the DRGs (Fig. S4 B, http://links.lww.com/PAIN/C330). Approximately 26.3% of them were A fibers (NF200^+^, Fig. S4 C, D, http://links.lww.com/PAIN/C330), and 53.7% were C fibers (Peripherin^+^, Fig. S4 E, F, http://links.lww.com/PAIN/C330), among which 34.5% were peptidergic (CGRP^+^, Fig. S4 G, H, http://links.lww.com/PAIN/C330) and 16% are nonpeptidergic (IB4^+^, Fig. S4 I, J, http://links.lww.com/PAIN/C330).

Finally, we counted 72.6 ± 10.9 cells per mm^2^ in the Ntng1-ChR2 line (Fig. S5 B, http://links.lww.com/PAIN/C330). Approximately 80.5% of ChR2-TdTomato^+^ cells were A fibers (NF200^+^, Fig. S5 C, D, http://links.lww.com/PAIN/C330) and 15.7% were C fibers (Peripherin^+^, Fig. S5 E, F, http://links.lww.com/PAIN/C330), with 1.9% being peptidergic (CGRP^+^, Fig. S5 G, H, http://links.lww.com/PAIN/C330) and 3.4% nonpeptidergic (IB4^+^, Fig. S5 I, J, http://links.lww.com/PAIN/C330).

In the spinal cord, the pattern of expression of ChR2-TdTomato was in line with what we found in the DRGs. Indeed, in the SNS-ChR2 line, the ChR2-TdTomato was mainly expressed in the superficial layers of the SCDH, corresponding to laminae I and II where most nociceptors enter (Fig. S4 A, http://links.lww.com/PAIN/C330). In the Advillin-ChR2 line, although we could see ChR2-TdTomato in the upper laminae, it was also expressed deeper in the SCDH, corresponding to the terminations of both nociceptors and nonnociceptors (Fig. S3A, http://links.lww.com/PAIN/C330). Finally, TdTomato in the Ntng1-ChR2 was not expressed in the substantia gelatinosa but only in deeper laminae of the SCDH, in line with the very low number of peptidergic and nonpeptidergic neurons expressing ChR2-TdTomato in the DRGs (Fig. S5 A, http://links.lww.com/PAIN/C330).

### 3.3. Nonnociceptive neurons are necessary but not sufficient to induce spinal microglial reactivity

**Figure 2.**
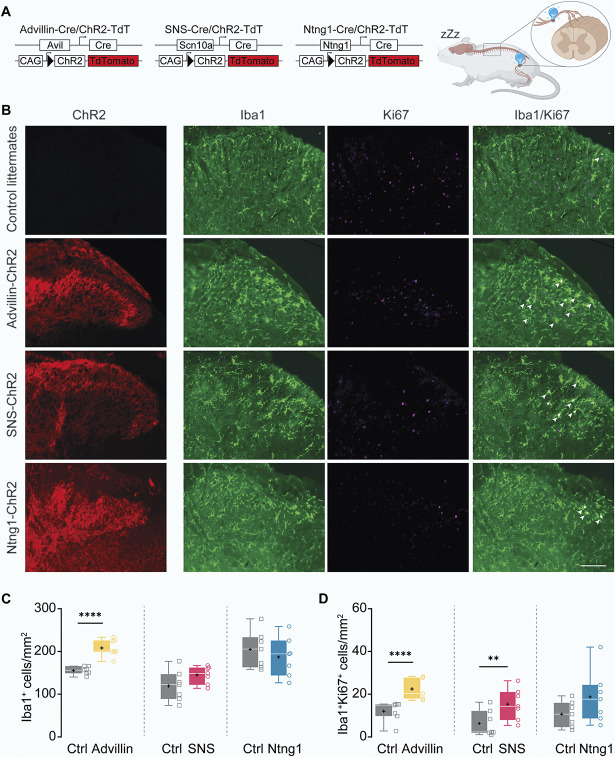
Optogenetic activation of combined nociceptive and nonnociceptive fibers is necessary to induce spinal microglial reactivity. (A) Experimental design. Construction of the 3 different transgenic line used and optogenetic stimulation. The stimulation was also applied on control littermates. Protocol was 2 Hz, 50 ms, 5 mW/mm^2^ for 30 minutes. (B) Representative images of ipsilateral SCDH 2 days after stimulation in control littermates, Advillin-ChR2, SNS-ChR2, and Ntng1-ChR2 mice (n = 7 per group) showing ChR2 (in red), Iba1^+^ cells (microglia, green), Ki67^+^ (proliferating cells, purple), and merged (proliferating microglia, in white). Arrowheads point to colocalization examples. Scale bar 100 μm. (C) Number of Iba1^+^ cells per mm^2^. Left (yellow). Mean ± SD: Ctrl = 155.2 ± 9.3, Advillin = 208.3 ± 20.1. 2W ANOVA (Dorsal horn side *P* < 0.0001, Group *P* = 0.0009, Interaction *P* = 0.0003) with Tukey multiple comparisons post hoc, *P* < 0.0001. Middle (pink). Mean ± SD: Ctrl = 120.7 ± 35.5, SNS = 146.5 ± 20.5. 2W ANOVA (Dorsal horn side *P* = 0.049, Group *P* = 0.2973, Interaction *P* = 0.1934). Right (in blue). Mean ± SD: Ctrl = 206 ± 43.3, Ntng1 = 188.7 ± 45.9. 2W ANOVA (Dorsal horn side *P* = 0.5543, Group *P* = 0.1311, Interaction *P* = 0.5799). (D) Number of Iba1^+^Ki67^+^ cells per mm^2^. Left (yellow). Mean ± SD: Ctrl = 12.1 ± 4.7, Advillin = 22.5 ± 5.2. 2W ANOVA (Dorsal horn side *P* < 0.0001, Group *P* = 0.0004, Interaction *P* = 0.001) with Tukey multiple comparisons post hoc, *P* < 0.0001. Middle (pink). Mean ± SD: Ctrl = 6.3 ± 6, SNS = 15.3 ± 7.3. 2W ANOVA (Dorsal horn side *P* < 0.0001, Group *P* = 0.0228, Interaction *P* = 0.0161) with Tukey multiple comparisons post hoc, *P* = 0.0082. Right (blue). Mean ± SD: Ctrl = 10.4 ± 6, Ntng1 = 18.5 ± 12.3. 2W ANOVA (Dorsal horn side *P* < 0.0001, Group *P* = 0.1288, Interaction *P* = 0.1361). Ctrl, control littermates.

Then, we optogenetically stimulated the left sciatic nerve of Advillin-ChR2, SNS-ChR2, and Ntng1-ChR2 mice (Fig. 2A). Control littermate animals were used as controls.

Two days after stimulation, we quantified Iba1 staining of spinal microglia and its colocalization with proliferation marker Ki67 (Fig. 2B). In line with electrical stimulation that recruits all types of sensory neurons, optogenetic activation of all sensory neurons using Advillin-ChR2 mice induced a strong increase in the number of Iba1^+^ microglial cells (Fig. 2C left, yellow, Ctrl = 155.2 ± 9.3 cells/mm^2^, Advillin = 208.3 ± 20.1 cells/mm^2^) and in the number of Iba1^+^Ki67^+^ cells (Fig. 2D left, yellow, Ctrl = 12.1 ± 4.7 cells/mm^2^, Advillin = 22.5 ± 5.2 cells/mm^2^) in ipsilateral SCDH compared to sham. No changes were observed in contralateral SCDH (see statistical table for values, http://links.lww.com/PAIN/C328).

Optogenetic activation of SNS-ChR2^+^ PSN induced a small increase in the number of Iba1^+^Ki67^+^ cells compared to sham (Fig. 2D middle, pink, Ctrl = 6.3 ± 6 cells/mm^2^, SNS = 15.3 ± 7.3 cells/mm^2^), but no increased number of Iba1^+^ cells was observed in ipsilateral SCDH between groups (Fig. 2C middle, pink, Ctrl = 120.7 ± 35.5 cells/mm^2^, SNS = 146.5 ± 20.5 cells/mm^2^).

Optogenetic activation of Ntng1-ChR2^+^ PSN did not induce an increase in the number of microglial cells (Fig. 2C right, blue, Ctrl = 206 ± 43.3 cells/mm^2^, Ntng1 = 188.7 ± 45.9 cells/mm^2^) or in the number of proliferating microglial cells compared to ipsilateral SCDH of control littermates (Fig. 2D right, blue, Ctrl = 10.4 ± 6 cells/mm^2^, Ntng1 = 18.5 ± 12.3 cells/mm^2^).

As for electrical stimulation, we did not observe any difference in the number of ATF3^+^ neurons in the averaged L3, L4, and L5 ipsilateral DRGs between the groups (Fig. S1 A, C-E, http://links.lww.com/PAIN/C330), indicating an absence of neuronal injury after optogenetic stimulation.

### 3.4. Spinal microglia change morphology in response to combined peripheral input from both nociceptive and nonnociceptive neurons

**Figure 3.**
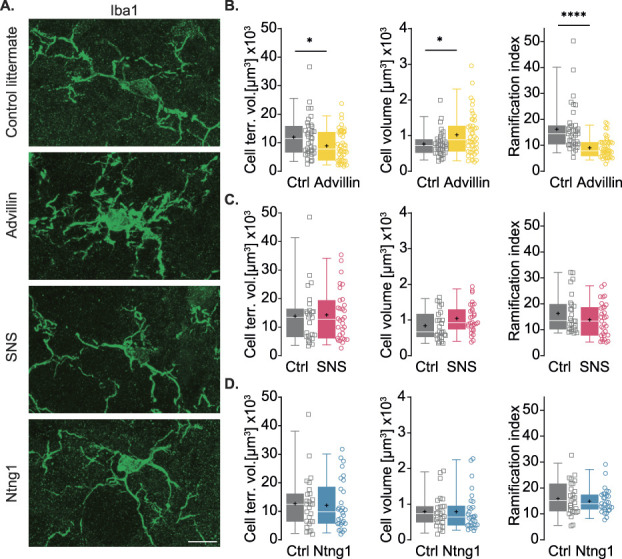
Activation of combined nociceptive and nonnociceptive fibers induces spinal microglia morphological changes. (A) Representative confocal images of Iba1^+^ cells in SCDH of control littermates, Advillin-ChR2, SNS-ChR2, and Ntng1-ChR2 after optogenetic stimulation. Scale bar 10 μm. (B) Quantification of microglial morphological features in control (n = 37 cells from 3 mice) and Advillin-ChR2 (n = 47 cells from 3 mice) mice. Mean ± SD (cell territorial volume): Ctrl = 11,963 ± 7001, Advillin = 8914 ± 5418, *P* = 0.0471. Mean ± SD (cell volume): Ctrl = 766.3 ± 344.3, Advillin = 1022 ± 591.4, *P* = 0.0471. Mean ± SD (ramification index) Ctrl = 16.2 ± 9.0, Advillin = 9 ± 3.8, *P* < 0.0001. Mann–Whitney test for all. (C) Quantification of microglial morphological features in control (n = 26 cells from 3 mice) and SNS-ChR2 (n = 31 cells from 3 mice) mice. Mean ± SD (cell territorial volume): Ctrl = 13,819 ± 9889, SNS = 14,274 ± 8739, *P* = 0.7206. Mean ± SD (cell volume): Ctrl = 840.2 ± 408.2, SNS = 1041 ± 402.8, *P* = 0.0678. Mean ± SD (ramification index) Ctrl = 16.3 ± 7.4, SNS = 13.8 ± 6.7, *P* = 0.1953. Mann–Whitney test for cell territorial volume, unpaired t test, 2-tailed for cell volume, and ramification index. (D) Quantification of microglial morphological features in control (n = 27 cells from 3 mice) and Ntng1-ChR2 (n = 30 cells from 3 mice) mice. Mean ± SD (cell territorial volume): Ctrl = 12,678 ± 9186, Ntng1 = 12,106 ± 8606, *P* = 0.7451. Mean ± SD (cell volume): Ctrl = 797.2 ± 465.2, Ntng1 = 788.8 ± 537.5, *P* = 0.6398. Mean ± SD (ramification index) Ctrl = 15.8 ± 6.4, Ntng1 = 14.9 ± 4.6, *P* = 0.7212. Mann–Whitney test for all. Ctrl, control littermates.

To further evaluate SCDH microglial reactivity, we also assessed their morphological changes (Fig. 3A) in response to the different peripheral inputs from PSN using the same MATLAB script used for electrical stimulation. Activation of nociceptive and nonnociceptive neurons in Advillin-ChR2 mice (Fig. 3B) induced a reduction in the cell territorial volume compared to control littermates (Ctrl = 11,963 ± 7001 μm^3^, Advillin = 8914 ± 5418 μm^3^). The overall cell volume was also increased (Ctrl = 766.3 ± 344.3 μm^3^, Advillin = 1022 ± 591.4 μm^3^). As a result, the ramification index (ratio between cell territorial volume and total cell volume) was significantly decreased (Ctrl = 16.2 ± 9.0, Advillin = 9 ± 3.8). No change was observed between SNS-ChR2 and control littermates (Fig. 3C) for cell territorial volume (Ctrl = 13,819 ± 9889 μm^3^, SNS = 14,274 ± 8739 μm^3^), cell volume (Ctrl = 840.2 ± 408.2 μm^3^, SNS = 1041 ± 402.8 μm^3^), and ramification index (Ctrl = 16.3 ± 7.4, SNS = 13.8 ± 6.7). Similarly, cell territorial volume (Ctrl = 12,678 ± 9186 μm^3^, Ntng1 = 12,106 ± 8606 μm^3^), cell volume (Ctrl = 797.2 ± 465.2 μm^3^, Ntng1 = 788.8 ± 537.5 μm^3^), and ramification index (Ctrl = 15.8 ± 6.4, Ntng1 = 14.9 ± 4.6) were unchanged in spinal microglia after activation of nonnociceptors in Ntng1-ChR2 compared to control littermates (Fig. 3D).

### 3.5. Combined activation of nociceptive and nonnociceptive neurons is necessary to induce pain hypersensitivity

**Figure 4.**
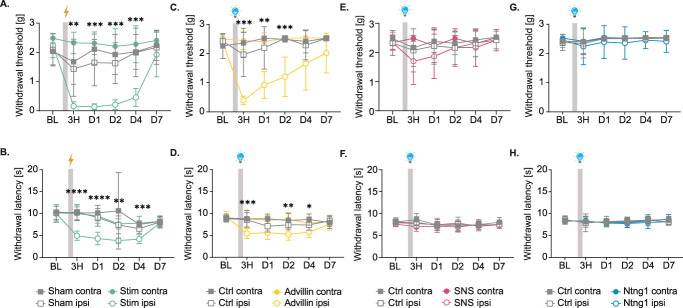
Activation of combined nociceptive and nonnociceptive fibers is necessary to induce pain hypersensitivity. Mechanical and thermal sensitivities assessed by measuring withdrawal threshold (Von Frey, A, C, E, G) and withdrawal latency (B, D, F, H) of ipsilateral and contralateral paws of mice before and after stimulation. (A and B) Sham (n = 10) vs Stim (n = 10) CX3CR1-eGFP mice. 2W RM ANOVA (Von Frey) Time (*P* < 0.0001, Group *P* < 0.0001, Interaction *P* < 0.0001). 2W RM ANOVA (Hargreaves) Time (*P* < 0.0001, Group *P* < 0.0001, Interaction *P* < 0.0001). (C and D) Advillin-ChR2 (n = 10) vs controls (n = 10). 2W RM ANOVA (Von Frey) Time *P* < 0.0001, Group *P* < 0.0001, Interaction *P* < 0.0001. 2W RM ANOVA (Hargreaves) Time (*P* < 0.0001, Group *P* < 0.0001, Interaction *P* < 0.0001). (E and F) SNS-ChR2 (n = 7) vs controls (n = 10). 2W RM ANOVA (Von Frey) Time *P* = 0.0357, Group *P* = 0.1119, Interaction *P* = 0.8197. 2W RM ANOVA (Hargreaves) Time *P* = 0.0246, Group *P* = 0.4750, Interaction *P* = 0.4547. (G and H) Ntng1-ChR2 (n = 10) vs controls (n = 10). 2W RM ANOVA (Von Frey) Time *P* = 0.1863, Group *P* = 0.1092, Interaction *P* = 0.9995. 2W RM ANOVA (Hargreaves) Time *P* = 0.1654, Group *P* = 0.4313, Interaction *P* = 0.9245. Comparisons shown are between ipsilateral paws of stimulated and of control groups for each timepoint. All ANOVA are 2W RM ANOVA with Geisser–Greenhouse correction, Tukey multiple comparisons post hoc. BL, baseline. Ctrl, control littermates. Contra, contralateral. Ipsi, ipsilateral.

We then investigated whether these stimulations would induce any long-lasting mechanical or thermal hypersensitivity. To do so, we repeated the electrical and optogenetic activation protocols. We measured mechanical and thermal sensitivity on both hind paws of animals using Von Frey and Hargreaves tests and compared the ipsilateral paw of the test groups to their contralateral paw and to their baseline (Statistical table, http://links.lww.com/PAIN/C328) and the ipsilateral paws of control groups (sham or control littermates, Fig. 4).

Electrical stimulation and combined optogenetic activation of nociceptive and nonnociceptive neurons with the Advillin-ChR2 mice line both produced hypersensitivity, whereas activation of SNS-ChR2^+^ or Ntng1-ChR2^+^ neurons, respectively modulating preferentially nociceptors or nonnociceptors, did not induce hypersensitivity.

Indeed, stimulated CX3CR1-eGFP developed mechanical hypersensitivity in the Von Frey test on their ipsilateral paw compared to the ipsilateral paw of sham (Fig. 4A). The withdrawal threshold from electrically stimulated mice was already significantly reduced 3 hours after the activation protocol compared to sham and persisted until 4 days after electrical stimulation. At day 7, it was back to baseline. Thermal hypersensitivity was also present after electrical stimulation (Fig. 4B). We found a significant decrease in withdrawal latency from 3 hours to day 4 after electrical stimulation. It was back to sham level at day 7.

Similarly, Advillin-ChR2 mice also showed mechanical hypersensitivity in the Von Frey test on their ipsilateral paw compared to the ipsilateral paw control littermates (Fig. 4C). Their withdrawal threshold was significantly reduced 3 hours after the optogenetic protocol. It was still significantly reduced until day 2. From day 4, the mechanical sensitivity was back to control littermate groups level. In the Hargreaves test, we also observed thermal hypersensitivity in Advillin-ChR2 compared to control littermates (Fig. 4D). We found a significant decrease in withdrawal latency on the ipsilateral paw at 3 hours, day 2 and 4 compared to control littermates but not at day 1. By day 7, the latency was back to the level of the ipsilateral paw of the control group.

In contrast, statistical analysis of mechanical sensitivity after optogenetic stimulation in SNS-ChR2 mice did not reveal any decrease of withdrawal threshold (Fig. 4E). The thermal sensitivity of SNS-ChR2 was also unchanged (Fig. 4F) compared to control littermates.

Optogenetic activation of nonnociceptors in Ntng1-ChR2 mice did not induce any changes in mechanical (Fig. 3G) or thermal sensitivity (Fig. 3H).

Comparison of the different lines to their baseline and their contralateral paw are shown in statistical table, http://links.lww.com/PAIN/C328.

### 3.6. Inhibition of microglia with minocycline prevents the development of hypersensitivity after electrical stimulation in males but not in females

**Figure 5.**
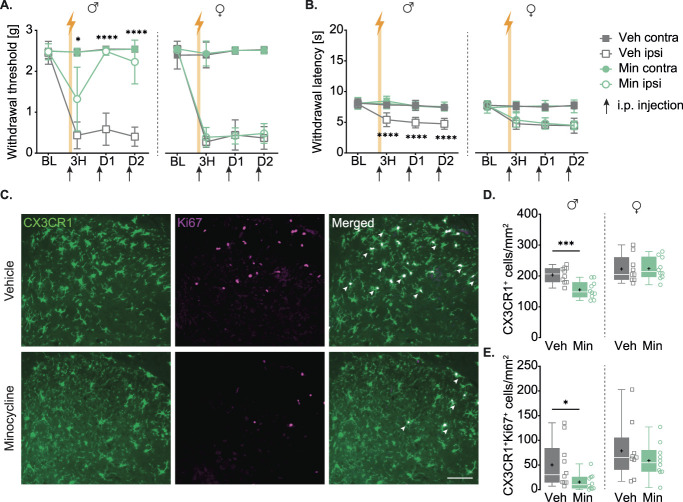
Minocycline prevents the development of hypersensitivity and SCDH microglial reactivity after electrical stimulation only in males. (A) Mechanical sensitivity of vehicle or minocycline treated group in males (left) and females (right). Comparisons shown are between ipsilateral paws of minocycline and vehicle groups for each timepoint. Males, 2W RM ANOVA with Geisser–Greenhouse correction (Time *P* < 0.0001, Group *P* < 0.0001, Interaction *P* < 0.0001). Tukey multiple comparisons post hoc. Females, 2W RM ANOVA with Geisser–Greenhouse correction (Time *P* < 0.0001, Group *P* < 0.0001, Interaction *P* < 0.0001). Tukey multiple comparisons post hoc. Arrows show injection timepoints. (B) Thermal sensitivity of vehicle or minocycline treated group in males (left) and females (right). Comparisons shown are between ipsilateral paws of minocycline and saline groups for each timepoint. Males, 2W RM ANOVA with Geisser–Greenhouse correction (Time *P* < 0.0001, Group *P* < 0.0001, Interaction *P* < 0.0001) Tukey multiple comparisons post hoc. Females, 2W RM ANOVA with Geisser–Greenhouse correction (Time *P* < 0.0001, Group *P* < 0.0001, Interaction *P* < 0.0001). Tukey multiple comparisons post hoc. Arrows show injection timepoints. (C) Representative images of males ipsilateral SCDH of vehicle and minocycline groups showing CX3CR1^+^ (microglia, in green), Ki67^+^ cells (proliferating, in purple), and merged (proliferating microglia, in white). Arrowheads point to colocalization examples. Scale bar 100 μm. (D) Number of CX3CR1-eGFP^+^ cells per mm^2^ in males (left) and females (right). Males, Mean ± SD: Veh = 203.2 ± 25.5, Min = 154.6 ± 27.7. 2W ANOVA (Dorsal horn side *P* = 0.0204, Group *P* = 0.0094, Interaction *P* = 0.0076) with Tukey multiple comparisons post hoc (*P* = 0.0019). Females, Mean ± SD: Veh = 222.5 ± 44.0, Min = 223.5 ± 35.5. 2W ANOVA (Dorsal horn side *P* = 0.0199, Group *P* = 0.4147, Interaction *P* = 0.4644). (E) Number of CX3CR1-eGFP^+^ Ki67^+^ cells per mm^2^ in males (left) and females (right). Males, Mean ± SD: Veh = 50.1 ± 47.1, Min = 15.5 ± 16.4. 2W ANOVA (Dorsal horn side *P* = 0.0002, Group *P* = 0.0321, Interaction *P* = 0.0373) with Tukey multiple comparisons post hoc (*P* = 0.0185). Females, Mean ± SD: Veh = 78.7 ± 58.1, Min = 59.1 ± 34.7. 2W ANOVA (Dorsal horn side *P* < 0.0001, Group *P* = 0.3797, Interaction *P* = 0.3625). Min, minocycline; Veh, vehicle.

To confirm the role of microglia in the effects we observed, we injected male mice with minocycline or vehicle (saline) and assess whether it could prevent spinal microglial reactivity and the development of mechanical and thermal hypersensitivity. We used the electrical stimulation protocol as it is our strongest model. Our results showed that minocycline could prevent a decrease in withdrawal threshold (Fig. 5A left) and latency (Fig. 5B left) compared to electrically stimulated mice that were injected with saline. We then investigated the effect of minocycline on SCDH microglial reactivity (Fig. 5C) by quantifying their number and proliferation. We found a decreased number of GFP^+^ cells in ipsilateral SCDH of mice injected with minocycline compared to vehicle group (Fig. 5D left, Veh = 203.2 ± 25.5 cells/mm^2^, Min = 154.6 ± 27.7 cells/mm^2^). Similarly, we also observed fewer Ki67^+^GFP^+^ cells in minocycline group compared to vehicle group (Fig. 5E left, Veh = 50.1 ± 47.1 cells/mm^2^, Min = 15.5 ± 16.4 cells/mm^2^), indicating a decreased proliferation in microglial cells. No changes were observed between the groups in the contralateral SCDH (see statistical table, http://links.lww.com/PAIN/C328).

Because spinal microglia have been shown to be sexually dimorphic in neuropathic pain models,^63^ we replicated this experiment in females. Electrical stimulation on the sciatic nerve in vehicle injected female mice induced a strong decrease in withdrawal threshold (Fig. 5A right) and latency (Fig. 5B right) compared to baseline (see statistical table, http://links.lww.com/PAIN/C328). In contrast to males, we found no difference in mechanical and thermal sensitivities between vehicle and minocycline groups. Similarly, we found no difference between the groups in the number of GFP+ cells in ipsilateral SCDH (Fig. 5D right, Veh = 222.5 ± 44.0, Min = 223.5 ± 35.5 cells/mm^2^) nor in the number of Ki67^+^GFP^+^ cells (Fig. 5E right, Veh = 78.7 ± 58.1, Min = 59.1 ± 34.7 cells/mm^2^).

## 4. Discussion

This study provides new insights into the differential contribution of distinct peripheral inputs to spinal microgliosis and long-lasting behavioral hypersensitivity in males, in the absence of any injury. Using electrical or optogenetic stimulations in different transgenic lines, our results revealed that broad activation of nociceptors and nonnociceptors was required to induce microgliosis, morphological and electrophysiological changes in microglia, and mechanical and thermal hypersensitivity without causing neuronal injury. In contrast, activation of nociceptor or nonnociceptors alone failed to produce spinal microglial reactivity and persistent pain. Our findings align with studies^30,79^ showing that recruiting only A fibers does not induce microglial reactivity and pain, whereas recruiting A and C fibers does. This is also consistent with research using nerve blocks^64,69,71^ or genetic ablation of nonnociceptive neurons after PNI.^58^ Another study demonstrated that optogenetic activation of nonnociceptive mechanoreceptors failed to induce persistent pain.^12^ Interestingly, our results contrast with a previous paper showing that activation of nociceptors alone induced pain lasting up to 24 hours.^17^ The discrepancy may be because of the different stimulation protocols, as their optogenetic stimulation was conducted on the hind paw rather than on the sciatic nerve, with different parameters. In addition, a study using a repeated cold stress protocol as a fibromyalgia model showed that activation of proprioceptors induced sustained pain and spinal microglial reactivity,^67^ although this model also upregulated ATF3 expression in proprioceptors, suggesting additional mechanisms. In our study, neither electrical nor optogenetic stimulation led to a significant ATF3 expression increase in the DRG neurons compared to controls, suggesting that the stimulation remains without notable nerve injury.

In rodents, NF200 has been extensively described as a marker for large, myelinated fibers (Aβ and Aδ),^24,38^ and Peripherin as a marker for unmyelinated small-caliber fibers (C fibers).^24^ Likewise, IB4 and CGRP staining have been useful in identifying nonpeptidergic and peptidergic unmyelinated fibers, respectively.^3^ As anticipated, the anatomical characterization of the transgenic mouse lines revealed that the Advillin-ChR2 line has a wide distribution among both nociceptive and nonnociceptive PSN. Although the SNS-ChR2 line does not seem to be entirely restricted to nociceptors, it is highly enriched in this specific population and widely used as a nociceptive transgenic line.^1,18,51^ This aligns with the new classification of PSN based on single-cell RNA sequencing, which shows that the SNS-Cre promoter, encoding sodium channel Na_v_1.8/9, is expressed in all nociceptors, including unmyelinated C fibers as well as in peptidergic myelinated fibers.^66^ Although this ensures broad targeting of nociceptors, it may also include a small fraction of mechanoreceptive fibers. Both the Advillin- and SNS-ChR2 lines exhibited a similar number of ChR2-positive neurons. However, only the stimulations in the first 1 led to a strong microglial reactivity and persistent pain. This suggests that the number of neurons per se is not the critical factor, rather, it is the proportion of each subpopulation of PSN. The characterization of the Ntng1-ChR2 line reveals high specificity toward nonnociceptive neurons, but with few ChR2-positive neurons in the DRG. For this mouse line, we cannot exclude that the lack of SCDH microglial proliferation is because of the low number of neurons being activated. Moreover, a difference in ChR2 expression efficiency between the 3 lines could partially explain the different results obtained. Of note, the nonnociceptive Ntng1-ChR2 line does not target the NF4 subtype of nonnociceptive neurons (see Table 1^58^). Therefore, we cannot extrapolate our findings to those specific subtypes of nonnociceptors. The role of NF4-type proprioceptive neurons could be evaluated by using a Cre line under the control of the parvalbumin promoter (Pvalb-Cre). Here, we used Cre lines to activate different populations of PSN: one predominantly targeting nociceptors, another selectively activating nonnociceptive subclasses, and a third broadly activating most PSN subtypes. It would be of interest to further refine our findings by using other Cre lines to gain insight into the role of each neuronal subtype in inducing SCDH microglial reactivity.

Earlier studies have shown that electrical stimulation of the sciatic nerve in naïve rats at a frequency recruiting Aβ, Aδ, and C fibers induced microgliosis^49^ and hypersensitivity until 2 days after stimulation. In contrast, electrical activation of Aβ/δ fibers alone failed to do so.^30^ In nerve injury models, a complete motor and sensory blockade of the sciatic nerve, but not a nociceptive block alone, prevented the development of chronic pain and spinal microglial reactivity.^64,69,71^ In the present study, we have demonstrated that the electrical stimulation of the sciatic nerve in mice elicited spinal microglial changes and provoked sustained behavioral hypersensitivity. It confirms previously described data in rats and further extends the spinal microglia reactivity description at electrophysiological and morphological level. Furthermore, our results indicate that the mechanical and thermal hypersensitivity, induced in mice by a similar brief electrical stimulation protocol, last for up to 4 days. Of importance, our data corroborate a link between microglia reactivity and sustained behavioral hypersensitivity in male mice. Indeed, minocycline prevented hypersensitivity and microglial proliferation. Interestingly, we found that microglial proliferation and behavioral hypersensitivity after electrical stimulation were also observed in female mice, and that minocycline failed to prevent these changes. This aligns with prior research indicating that microglia play a prominent role in pain sensitization in males, whereas alternative immune mechanisms, such as T cells, may be more relevant in females.^62,63^ The absence of a significant inhibitory effect of minocycline in female mice emphasizes the need for further investigations to uncover sex-specific mechanisms underlying microglial reactivity and pain chronification.

Notably, our stimulations did not increase proliferation in peripheral macrophages, contrasting with observations in PNI models of neuropathic pain,^36,37,78^ suggesting distinct mechanisms for macrophage and SCDH microglia reactivity.

We postulated that spinal microglia changes upon peripheral stimulation recapitulate the pathological phenotype. Our previous studies revealed that spinal microglia massively proliferate 2 days after spared nerve injury (SNI) and show a strong increase in number at days 4 and 7 post-SNI, both in mice and rats.^22,23^ Similarly, in the present study, we found that the brief electrical stimulation triggered a strong increase in microglia number 2 days later and enhanced proliferating microglia. Using a refined optogenetic stimulation, we observed a similar histological result only with the combined activation of nociceptive and nonnociceptive inputs. Furthermore, previous studies have shown that spinal microglia exhibit significant electrophysiological changes upon nerve injury, which was suggested to relate to their inflammatory and proliferating profile.^23^ Accordingly, we found that brief electrical stimulation is sufficient to hyperpolarize the resting membrane potential and increase inward current densities in microglia. Spinal microglial morphological changes have been broadly described under PNI conditions.^11,35,39,73^ Our results showed that combined nociceptive and nonnociceptive stimulations, via broad electrical or optogenetic approaches, significantly decreased the ramification index and increased cell volume. Qualitatively, these microglia exhibit shorter, beaded processes, which could be characteristic of a hypertrophic or dystrophic morphology, both associated with pathological states.^59^

There are several limitations of this study, which can be addressed in future studies. Minocycline was used to inhibit microglia, and it is known to act on other cell types.^50^ Thus, the use of a more specific inhibitor could allow to further confirm the link between spinal microglial reactivity and pain hypersensitivities after stimulations of the PSN. In addition, we cannot exclude that our stimulation parameters may not have optimally targeted the intended neuronal subpopulations. Nonetheless, based on anatomical characterizations and behavioral and microglial responses, our results support the conclusion that broad activation of nociceptors and nonnociceptors induces sustained pain and SCDH microglial reactivity, through mechanisms that remain to be elucidated. Although extensive research has identified numerous neurotransmitters and neuropeptides specific to nociceptors such as CGRP, Substance P (SP), and Adenosine triphosphate (ATP),^4,8,60^ to our knowledge, no neuropeptides or transmitters specific to nonnociceptive neurons have been identified. Substance P, released by nociceptive neurons, has been shown to potentiate glutamate excitatory transmission.^20,42,55^ Alternatively, combined activation of the 2 types of sensory neurons could lead to the secretion of yet unidentified factors, such as miRNAs. Interestingly, the spinal excitatory postsynaptic currents, induced by optogenetic activation of PSN axonal terminals, are potentiated by the perfusion of the miRNA let-7b. Let-7b is enhanced within the DRGs and SC upon peripheral inflammation and is potent to provoke a microgliosis.^15^ Similarly, miRNA-16-5p has been identified in the DRGs exosomes and shown to promote spinal microglia activation, which in turn contribute to neuropathic pain behavioral phenotype.^72^ These results thus suggest that a transfer of miRNAs from neurons to immune cells occurs. Understanding whether nonnociceptors release unidentified factors, or whether a combined release of factors through both populations would potentiate each other, will be crucial for elucidating the mechanism behind SCDH microglial reactivity and sustained pain.

Blocking nociceptor activity alone has shown to not be sufficient to prevent or reduce neuropathic pain induced by injury in murine models. Similarly, in clinical practice, nerve blocks targeting nociceptive fibers to prevent chronic postoperative pain have shown mixed results,^65^ likely because of the insufficiency of blocking nociceptive activity alone. Our data suggest that the combined activation of nociceptive and nonnociceptive fibers is responsible for spinal microglial reactivity and persistent pain. Understanding these mechanisms could lead to better therapeutic strategies to prevent chronic postoperative pain.

## Conflict of interest statement

Authors report no conflict of interest.

## Supplemental digital content

Supplemental digital content associated with this article can be found online at http://links.lww.com/PAIN/C328, http://links.lww.com/PAIN/C330.

## Supplemental video content

A video abstract associated with this article can be found on the PAIN Web site.
